# Extensive molecular differences between anterior- and posterior-half-sclerotomes underlie somite polarity and spinal nerve segmentation

**DOI:** 10.1186/1471-213X-9-30

**Published:** 2009-05-22

**Authors:** Daniel ST Hughes, Roger J Keynes, David Tannahill

**Affiliations:** 1 Department of Physiology, Development and Neuroscience, University of Cambridge, Downing Street, Cambridge, CB3 2DY, UK; 2 The Wellcome Trust Sanger Institute, Hinxton, Cambridge, CB10 1SA, UK; 3 Cranfield Health, Cranfield University, Cranfield, Bedfordshire, MK43 0AL, UK

## Abstract

**Background:**

The polarization of somite-derived sclerotomes into anterior and posterior halves underlies vertebral morphogenesis and spinal nerve segmentation. To characterize the full extent of molecular differences that underlie this polarity, we have undertaken a systematic comparison of gene expression between the two sclerotome halves in the mouse embryo.

**Results:**

Several hundred genes are differentially-expressed between the two sclerotome halves, showing that a marked degree of molecular heterogeneity underpins the development of somite polarity.

**Conclusion:**

We have identified a set of genes that warrant further investigation as regulators of somite polarity and vertebral morphogenesis, as well as repellents of spinal axon growth. Moreover the results indicate that, unlike the posterior half-sclerotome, the central region of the anterior-half-sclerotome does not contribute bone and cartilage to the vertebral column, being associated instead with the development of the segmented spinal nerves.

## Background

The subdivision of embryonic tissues into serial repeat-units, or segments, is a fundamental patterning process in early vertebrate development, and is most prominent in the formation of the mesodermal somites. Somites arise as paired epithelial spheres that bud off from the undifferentiated paraxial mesoderm (presomite mesoderm, PSM) flanking the notochord, and subsequently give rise to several tissues including the segmented axial skeleton and epaxial musculature of the adult organism [1,2].

**Figure 1:**
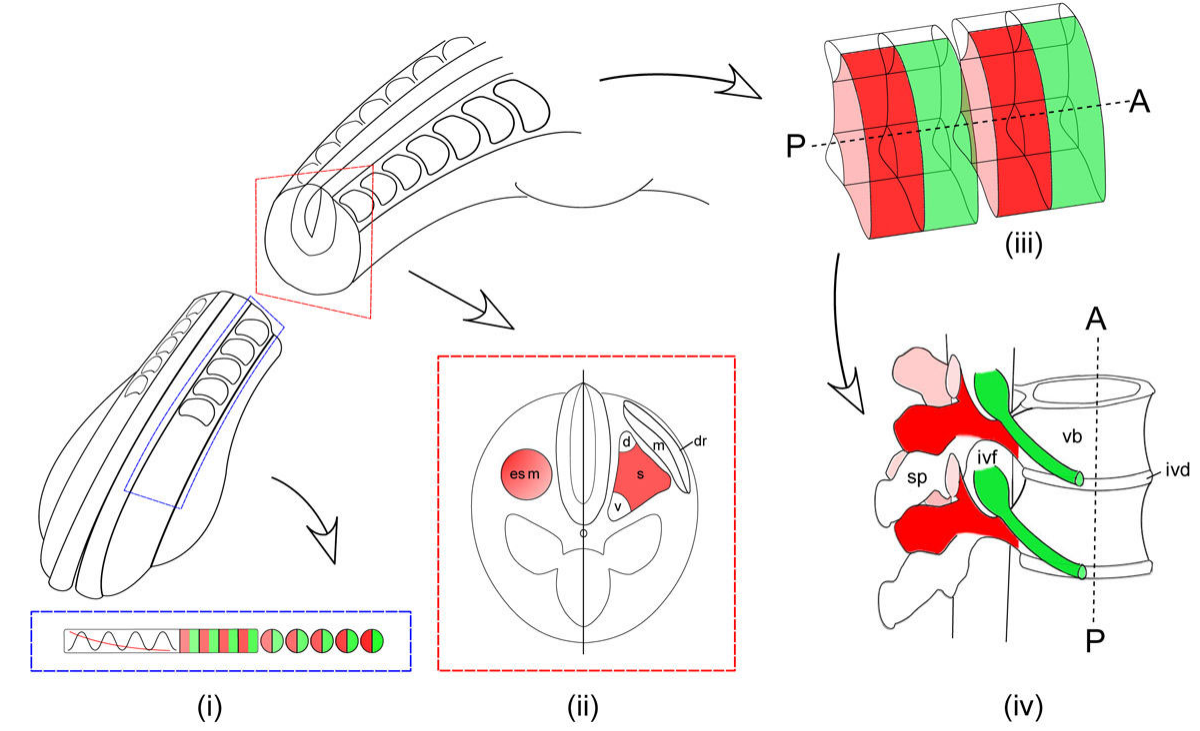
**Somite patterning and fate**. Somite development involves two patterning systems operating along the A-P and D-V axes. (i) Unsegmented presomite mesoderm and nascent somites showing the oscillations and gradients of gene activity that determine A-P polarity prior to overt somite formation (green: anterior half-somite; red: posterior half-somite). (ii) Transverse section through an A-half-epithelial somite (esm, left) and a differentiated somite (right). Patterning along the D-V axis sub-divides the somite into dermatome (dr), myotome (m) and sclerotome (s). The sclerotome is further sub-divided into ventral (v), central (s, red) and (d) dorsal regions. (iii) Representation of two somites viewed laterally, showing the central sclerotome A-P sub-division. Only the anterior-half (green) is permissive for PNS components. (iv) In differentiated vertebrae, posterior-central sclerotomes form the paired transverse processes and pedicles of the neural arches (red) that encase the spinal cord and provide attachment points for epaxial muscles. Anterior central-sclerotome derivatives (green) contribute to peripheral nerve sheaths and prefigure the positions of the intervertebral foraminae (ivf). Spinous process (sp), intervertebral disc (ivd), vertebral body (vb).

The formation and development of somites involves the superposition of two orthogonal patterning systems acting within the paraxial mesoderm. The first operates along the anterior-posterior (A-P) axis and is intrinsic to the paraxial mesoderm, resulting in individuation of the somites and their concomitant polarization into anterior and posterior halves (Figure 1). Somite formation is dependent on a molecular 'clock' within the PSM that generates periodic expression of genes including the notch, wnt and fibroblast growth factor (FGF) signalling pathways [3-5]. These cyclical gene expression patterns are superimposed on a regressing, longitudinal gradient of FGF expression along the A-P axis that results in the co-ordinated maturation of groups of PSM cells into each successive somite. Somite polarity is determined within the anterior PSM by a complex feedback mechanism mediated by notch signalling, the transcription factors tbx6 and mesp2, and ripply2, a negative regulator of mesp2 [6-11].

A second patterning system operates after somite formation, within both dorso-ventral and medio-lateral axes, and involves the early antagonistic and synergistic action of signalling molecules, such as sonic hedgehog, noggin and wnt, that originate outside of the paraxial mesoderm [12-15]. This separates the somite into two major sub-components, the sclerotome (skeletal/vertebral precursor cells) and the dermamyotome (muscle and dermis precursors). It also subdivides the sclerotome into further regions that are distinguished in terms of both morphogenesis and gene expression (Figure 1). These include the ventral peri-notochordal sclerotome that forms the vertebral bodies and intervertebral discs; the central (mid-dorso-ventral) sclerotome that generates the vertebral pedicles, transverse processes and most of the neural arches; and the dorsal sclerotome that gives rise to the vertebral spinous processes [2,12,13]. In keeping with the differing morphogenesis of these regions, their chondrogenesis is controlled by distinct upstream mechanisms [12,13]. For example, central sclerotome differentiation is regulated by *uncx4.1*, and *uncx4.1*-deficient mice exhibit a complete loss of the central sclerotome-derived vertebral components mechanisms, while ventral sclerotome is independently regulated by *pax1 *and *pax9*, thus ventral development is unperturbed in *uncx4.1 *mutants [16-18].

The co-ordinated action of these patterning systems creates a set of volume elements in the sclerotome that generate the mature axial skeleton via a process termed resegmentation [19-22]. The alternating arrangement of A- and P-ventral-half-sclerotomes pre-configures serial repetition of the vertebral bodies and intervertebral discs, and generates a weight-bearing yet flexible vertebral column that supports the head and trunk. The A- and P-central half-sclerotomes form the vertebral canal, which houses and protects the spinal cord, and molecular differences between the two halves of the sclerotome are critical for this process. The P-central-half-sclerotomes, which form the walls of the canal (vertebral pedicles, transverse processes and neural arches), also impart segmental barriers to emigrating neural crest cells and spinal axon growth cones, thereby imposing secondary segmentation on the developing peripheral spinal nerves [23-28]. By contrast, the A-central-half-sclerotomes form a permissive environment in which neural crest cells can differentiate to form the segmental dorsal root ganglia, and through which further neural crest cells, as well as motor and primary sensory axons, can navigate. The A-central-half-sclerotomes ultimately yield the intervertebral foraminae, ensuring that the spinal nerves can enter and exit the spinal cord unimpeded by the walls of the vertebral canal; they also contribute cells to the endoneurium and perineurium of the spinal nerves [2,12,29]. In sum, somite polarity ensures optimum mechanical protection of the spinal cord while simultaneously permitting movement of the vertebrate trunk and bidirectional nervous transmission between the spinal cord and the periphery. Recently, another functional role of A-P somite polarity has been uncovered, as definitive aortic endothelial cells have been shown to originate from the P-half-somite [30].

While the normal development and function of the vertebrate trunk is critically dependent on correct somite polarity, it remains unclear how the molecular differences between A- and P-cells are transcriptionally-regulated. Moreover, while a substantial degree of molecular complexity is likely to distinguish A- from P-cells, and several such differences have been documented, the detailed molecular components and their interactions are unknown [25,27]. Identifying such differences is a prerequisite for a sophisticated understanding of vertebral column genetics and the genesis of vertebral anomalies, as well as for future stem cell and tissue engineering strategies to repair the injured or diseased vertebral column. As a first step towards defining the full range of the molecular differences between A- and P-half sclerotomes, we have adopted a novel gene expression-profiling strategy based on the analysis of expression microarrays of micro-dissected mouse sclerotome components. Our results highlight the degree to which somite polarity at the cellular level is reflected by extensive molecular differences between the two somite halves.

## Results

**Figure 2:**
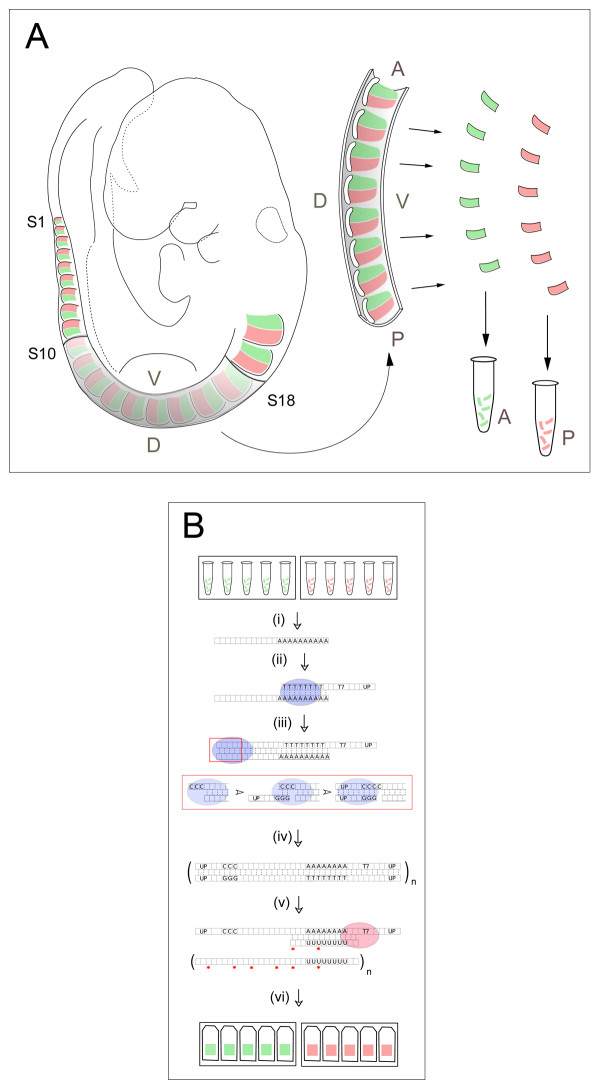
**(A) Half-sclerotome dissection**. Strips corresponding to the 10–18^th ^most recently formed somites (S10-S18) were isolated from mouse embryos at TS15 (9.5–10.25 d.p.c.). Individual A- (green) or P-sclerotome (red) halves were dissected and pooled for further processing. V, ventral; D, dorsal; A, anterior; P, posterior. **(B) Amplification of sclerotome RNA**. (i) Total RNA was purified individually from pools of A- or P-half-sclerotomes. (ii) First strand cDNA was synthesized by reverse transcriptase (blue) using a modified Clontech SMART primer containing oligo (dT), a T7 RNA polymerase promoter and an universal primer (UP). (iii) The template-independent addition of 3' C residues allows second strand synthesis by strand-switching of reverse transcriptase using a second UP bearing G residues. (iv) Global cDNA amplification by limited PCR cycles using a UP alone. (v, vi) Further amplification, and generation of antisense-labelled probe by *in vitro *transcription using T7 polymerase (red) for hybridization to microarrays

To identify novel genes involved in the establishment and maintenance of the anterior-posterior (A-P) difference between the two halves of the sclerotome, we adopted a microarray-based expression-profiling approach. Pilot experiments indicated that ~300 pg of total RNA could be isolated per sclerotome half (estimated as ~150 cells). Multiple sclerotome-halves were dissected as described in the Methods and pooled as depicted in Figure 2A. The pooling strategy was used for two reasons: first, to avoid inefficient amplification of sub-nanogram quantities of RNA, and second, to minimize the variation in gene expression that might be caused by position-specific and somite-stage-specific differences in the samples.

To generate sufficient material for profiling by microarray, a global amplification method was designed to overcome the detrimental effects of high-cycle-number PCR amplification on transcript representation, and to alleviate inefficient amplification by *in vitro *transcription in the nanogram range (Figure 2B) [31]. This method employs an initial low-cycle-number PCR amplification using strand-switching PCR amplification approaches, followed by linear *in vitro *transcription and subsequent labelling of the amplified population. Amplified RNA was hybridised to the mouse genome 430 2.0 Genechip (Affymetrix) as described in the Methods. The raw image files and computed intensity data are available from ArrayExpress (accession number: E-MEXP-913 . To ensure the efficacy of downstream analysis, all array data were subjected to quality control steps prior to further analysis (see Methods).

### Differential gene expression in somites

**Figure 3:**
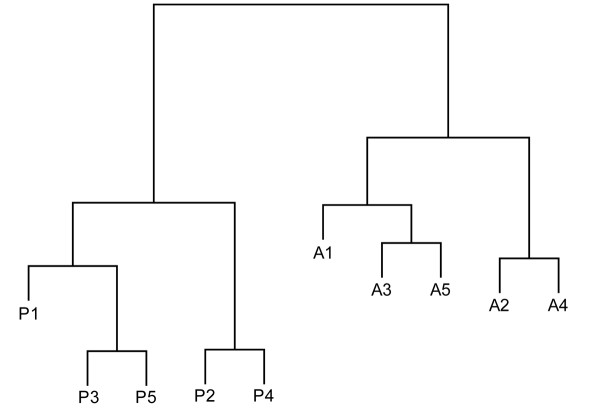
**Clustering of individual arrays with respect to genes known to shown differentially-expressed in A- or P-half-sclerotomes**. Dendrogram of agglomerative hierarchical clustering of individual array hybridization experiments based upon expression data for genes previously reported to show differentially expression between A- and P-sclerotome-halves. The arrays cluster into two distinct groups, and confirm the original identity of the dissected material as A- or P-half-sclerotome.

To confirm that the embryo dissection and subsequent processing had accurately generated anterior- and posterior-half-sclerotome populations without significant cross-contamination, we analysed the microarray data for expression of genes that have been shown unequivocally in previous studies to be differentially-expressed between A- and P-half-sclerotome (see [25] for general review). The expression measures within the array data for 11 known differentially-expressed genes (*efnb2, ephb3, fgf3, fgfr1, meox1, nmyc1, sema3a, sema3f, spon1, tbx18, uncx4.1*: see Additional File 1) were used for agglomerative hierarchical clustering of the arrays within the R statistical environment (see Methods). This procedure makes no assumptions about the identity of individual arrays as anterior or posterior and allows monitoring of the relatedness of the arrays to each other. The dendrogram in Figure 3 resulting from this analysis shows that each array separates into one of two distinct groups corresponding to its origin as A- or P-half-sclerotome. This pattern is distinct from that generated by clustering all genes on the array, and from that generated by clustering selected probe data sets that are not differentially-expressed (see Additional File 2). Overall, these results indicate that all A-half-sclerotome samples are more closely related to all other such samples, and that all P-half-sclerotome samples are likewise more closely related to each other, so any cross-contamination is likely to be insignificant. Differences between the two groups of array data are likely, therefore, to reflect significant molecular differences between A- and P-sclerotome-halves.

**Figure 4:**
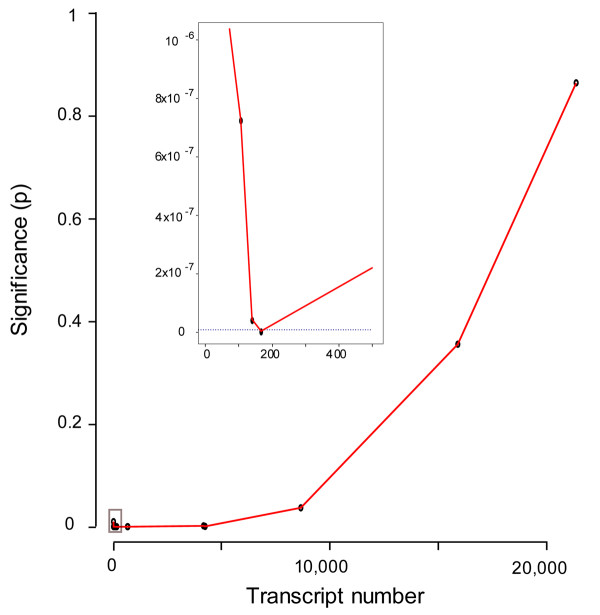
**Statistical significance of differential gene expression in the array data**. Fisher's exact test was used to explore the null hypothesis that known differentially-expressed genes are not related to their expression values in A- versus P-half-sclerotome. The y-axis shows the probability (p) that a pool of transcripts shows no enrichment of differentially-expressed genes; the lower the p-value the more likely it is to have significant enrichment. The x-axis shows the rank-ordered pool size of transcripts as determined by statistical certainty of differential expression in ascending order. The inset shows an expanded view of the rectangle indicated close to the origin. The dotted line corresponds to p = 10^-9^. The maximum statistical significance, and thus the minimum estimate for the total number of genes that are differentially-expressed between the two sclerotome halves, is obtained with a pool of ~175 transcripts.

Having confirmed the distinct anterior and posterior identity of the dissected samples, we estimated next the total number of genes differentially-expressed between anterior- and posterior-sclerotome-halves. We first ranked transcripts in terms of statistical certainty of differential expression between A- and P-halves using Welch's t-test, and then examined the transcript distribution of the 11 known differentially-expressed genes described above. While many of these transcripts are evenly distributed across the rank-ordered transcript range, it is notable that more than one third are found at the low end of the p-value distribution, and that this is not observed with non-differentially-expressed somite-related genes (Additional File 3). This suggests that our array analysis has a high probability of revealing differential expression. Furthermore, visual inspection of the rank-order (Additional File 1) suggests that there are as many as 700 genes with a high probability of differential expression. To provide a more accurate estimate of the total number of differentially-expressed genes and additional statistical validation, Fisher's exact score was used to determine the significance of enrichment of differentially-expressed genes within increasing group sizes amongst the rank-ordered transcripts relative to the genome as a whole. Figure 4 demonstrates that the lowest p-value, which provides a minimum estimate of the total number of differentially-expressed genes between the anterior and posterior sclerotomes halves, corresponds to ~175 transcripts. Further examination of this graph (Additional File 4) indicates again that there may be as many as ~650 differentially-expressed genes, as p-values rise very rapidly for pool sizes greater than 650 (see Additional Files 5 and 6 for lists of transcripts 1–175 and 176–850 respectively).

Of the 175 genes, we find that 57 (32.6%) are expressed in the A-half-sclerotome while 118 (67.4%) are expressed in the P-half-sclerotome. The relative proportions of A- and P- specific expression are in keeping with estimates from the literature suggesting that around 20 markers are differentially-expressed between A- and P-sclerotome with ~75% showing P-specific expression [25,32]. Overall, this indicates that there are significant transcriptional differences between the two somite halves and also reveals a posterior bias of differential transcriptional activity between the A- and P-sclerotome-halves (see Discussion). Our data further indicates that the total number of differentially-expressed genes may be some 8–30-fold higher than previously reported.

### Functional categorisation of candidate genes

To assess whether there are particular molecular processes significantly associated with either the A- or P-half-sclerotome genes, the Database tool for Annotation, Visualization and Integrated Discovery was used with Gene Ontology Consortium terms (GO, ), biochemical pathways (Kyoto Encyclopedia of Genes and Genomes, KEGG, ) and protein domain family names (PFAM, ). Additional File 7 lists all resulting terms for biological processes that show a significant probability of enrichment relative to the whole genome for the first 175 genes. While we have focused our discussion on statistically most reliable (p < 10^-3^) and therefore necessarily more general categories in the ontological hierarchies, Additional File 7 presents an extended list of terms from p < 10^-1^. Of note is the finding that there is nearly 2-fold enrichment within the P-half-sclerotome for expression of 'nuclear proteins' (GOTERM_CC_ALL nucleus: 30.4%, p = 9.11 × 10^-5^), much of which appears attributable to >2-fold over-representation of 'transcriptional regulators' (GOTERM_BP_ALL regulation of transcription: 20%, p = 2.94 × 10^-4^). Other enriched GO terms in the P-half include ~2-fold enrichment in genes associated with 'metabolic regulators' (21.6%, p = 2.60 × 10^-4^), 'regulation of cellular physiological processes' (25.6%, p = 2.06 × 10^-4^) and 'nucleic acid binding' (24.8%, p = 1.79 × 10^-4^). A similar comparison of PFAM families (SP_PIR_KEYWORDS) supports such findings and also reveals several additional enriched categories in the P-half-sclerotome, including phosphorylation, showing a 3-fold enrichment (16%, p = 2.07 × 10^-5^), 'developmental protein', showing a 4.7-fold enrichment (8%, p = 2.53 × 10^-4^) and 'membrane proteins' with a ~1.8-fold enrichment (18.4%, p = ~5 × 10^-3^). In comparison with the P-half-sclerotome, the A-half-sclerotome shows little enrichment of functional categories with p < 10^-3^. Only the GO term 'regulation of cellular process I' is found with a 2.3-fold over-representation (24%, p = 8.64 × 10^-4^). It is of interest, from casual inspection of probabilities > 10^-3^, that there is a posterior enrichment of genes involved in 'axon guidance', as well as 'UDP-glycosyltransferase activity' which is required for axon repulsion mediated by P-half-sclerotome cells [23]. As the number of David IDs associated with these terms is small some caution must be made in interpretation for less stringent p-values.

**Figure 5:**
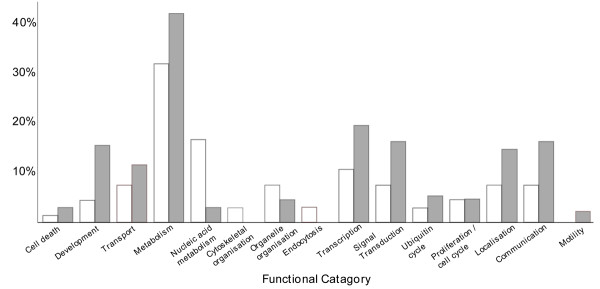
**Functional categories of A- and P-sclerotome-half restricted genes**. Each A- or P differentially-expressed gene was placed into one of fifteen functional categories based on BLAST and GO database descriptors (grey, P-restricted genes; white, A-restricted genes). A higher proportion of P-half-sclerotome restricted genes fall into functional categories such as development, signal transduction and transcriptional regulation.

While the above analysis compares gene expression in the A- and P-half-sclerotomes relative to the whole genome, we also undertook a direct comparison of gene expression in A- versus P-half-sclerotome. A combination of BLAST  searches and the GO database was used to assign a functional term to each of the 175 genes that are differentially-expressed between the two sclerotome halves. These terms were then grouped into 15 different categories (Figure 5). Again, this reveals a higher proportion of genes involved in transcription expressed in the P-half-sclerotome relative to the A-half (19.4% versus 10.6%). Furthermore, the number of P-half-enriched genes involved in signal transduction is more than double that for A-half-enriched genes (16.3% versus 7.6%) and include two members of the *wnt *family (*wnt5a, nkd2 homolog*), two *tgf *pathway genes (*tgfbr2*, *bmp5*) and two *mapk *pathway genes (*mapk8, atf2*). Figure 5 also shows that there are more than three-fold as many genes with known developmental functions expressed in the P half-sclerotome compared with the A-half. In keeping with a bone/cartilage fate for the P-half sclerotome, it is notable that we find selective expression in the P-half of the master regulator of chondrogenic differentiation, *sox9 *[33], as well as *bmp5*, a member of the bone morphogenetic protein family.

### Further confirmation of differential gene expression

Of the 11 known differentially-expressed genes, 6 (*uncx4.1, meox1, nmyc1, tbx18, sema3a, efnb2*) are found within the top 175 genes in our list. This analysis has revealed many more differentially-expressed genes, for which we sought independent confirmation by whole-mount *in situ *hybridisation (WISH). Within the list of 175, genes were prioritized according to the significance of their differential expression, using both Significance Analysis of Microarrays (SAM) with a false discovery rate of 10%, and the Bayesian Cyber-T approach, with p < 10^-4^. The original Affymetrix probe sequences were obtained from NetAffx , and BLAST was used to map sequences to Ensembl gene identifiers using the Ensembl genome-browser . After cropping the list of ambiguous and redundant entries, a revised list containing 50 entries was generated. Additionally, the same analysis was applied to the genes in the range 176–850, the region over which enrichment remained statistically highly significant, and generated a further list of 15 genes.

**Figure 6:**
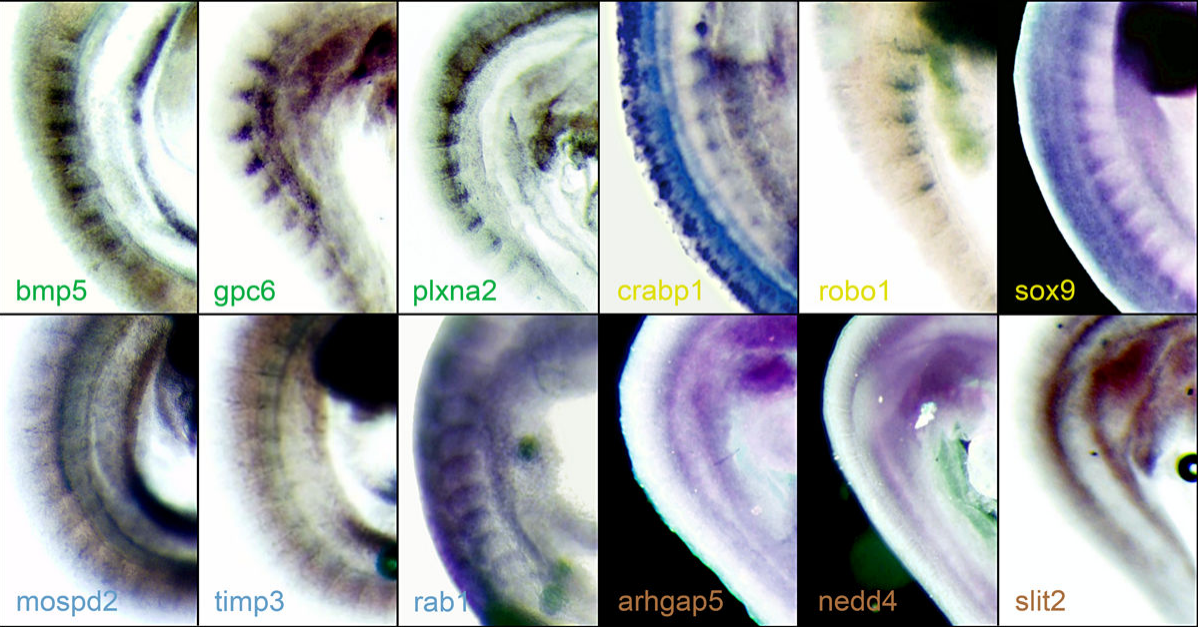
**Whole-mount *in situ *hybridization of candidate genes differentially-expressed between A- and P-half-sclerotome**. 56 candidate genes were tested for differential expression in A- and P-sclerotome-halves by whole-mount *in situ *hybridization. The resultant expression patterns were placed into four different groups based upon their certainty of differential expression. Groups 1–3 represent genes with decreasing certainty of differential expression (group 1, high certainty; group 3, low certainty), while group 4 genes show no apparent staining or no observed differential expression. Three examples from each group are shown: Group 1: *bmp5, gpc6, plxna2 *(green text)*; *Group 2: *crabp1, robo1, sox9 *(yellow text); Group 3: *mospd2, timp3, rab1 *(blue text); Group 4: *arhgap5, nedd4, slit2 *(brown text). Table 1 and Additional File 8 present additional data for the remaining 44 genes.

Overall, we generated 41 (82%) WISH probes from the top 175 genes and 15 (100%) probes from the next 176–850 genes. *In situ *hybridisation was performed on TS15-17 (9.5–10.5 d.p.c.) embryos. The results for the top 175 candidates were classified into 4 groups based upon somite staining pattern: Group 1, unambiguous differential expression (7 genes, 17%); Group 2, very likely to be differentially-expressed (8 genes, 19.5%); Group 3, likely to be differentially-expressed (10 genes, 24.4%); and Group 4, non-staining or no differential expression (16 genes, 39%); see Figure 6, Table 1, Additional File 8. The equivalent results for the 176–850 genes are as follows: Group 1 (2 genes, 13.3%), Group 2 (2 genes, 13.3%), Group 3 (2 genes, 13.3%), Group 4 (9 genes, 60%). This shows that even within the range of candidates ranked from 176–850, we can still detect a similar fraction of Group 1 genes (unambiguous differential expression) compared with the candidates ranked from 1–175. In summary, considering a candidate list from 1–850 as a whole, revalidation of the microarray candidates by *in situ *hybridisation indicates a positive rate of 55.4% for groups 1, 2 and 3 inclusive.

**Table 1:** Classification of candidate differentially-expressed sclerotome genes as assessed by whole-mount *in situ *hybridization

**Group 1**	**Group 2**	**Group 3**	**Group 4**
bmp5, flrt2 gpc6, meox1 nmyc1, plxna2 uncx4.1 [igfbp5 (766) lef1 (297)]	efnb2 acpl2/nm153420 mtdh, rbp1, robo1 rps6 sema3a sox9 [crabp1 (323) fap (178)]	anapc4/d5Ertd249e, ets2 fgfr1op, gpr150 mospd2/2410013I23rik rab1, rab28, timp3, tpr/C77892 trappc6b [cd44 (535) Q8C5E4/9430095K15rik (243)]	apg5l, arhgap5, btc cnot7, d17wsu104e lxn, narg1, nedd4 nm028130/2610020C11rik enh/1110001A05rik, nm027740 q8cak8, st13/3110002K08rik tbc1d24, tcfap2b zc3h6/4631426G04rik [slit2 (851), cd44 (535) cdc42 (337), col4a5 (271) dcc (647), ddx3y (344) eif4e (395), galgt1 (491) hexb/a930009M04rik (645)]

Expression patterns were classified into 4 groups based upon somite staining: Group 1 (unambiguous differential expression), Group 2 (likely to be differentially-expressed), Group 3 (probably differentially-expressed), and Group 4 (non-staining or no differential expression); Results from the 1–850 candidate range are shown. Genes in the range 176–850, with their respective ranking, are separated by a space and enclosed by square brackets (see Figure 6 and Additional File 8 for whole-mount *in situ *hybridization images for each gene).

**Figure 7:**
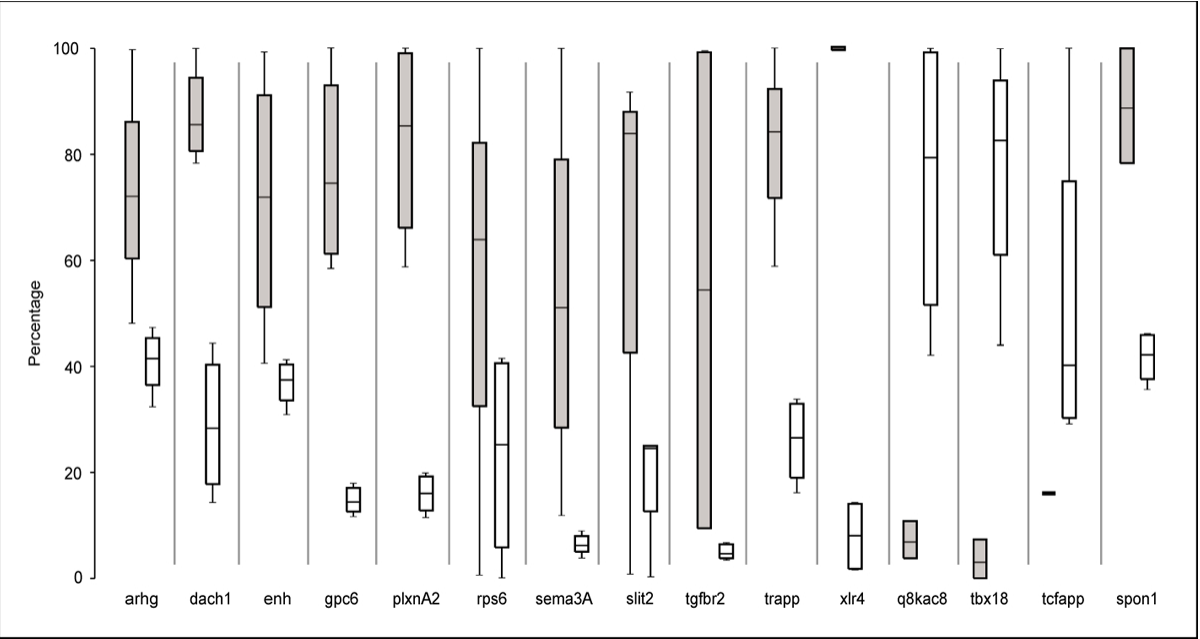
**qPCR analysis of differential expression between sclerotome halves**. qPCR was used to assess the relative expression in A- and P-half-sclerotomes of a selection of candidate genes identified from array and ISH experiments. Data for 11 genes with P-half sclerotome enrichment, 3 with A-half enrichment and spondin-1 as a P-half control are shown. Box plots indicate dispersion and skewness of the numerical distribution of expression values. qPCR values from individual experiments were expressed as a fraction of the highest value obtained for that gene in each half-sclerotome. No shading and grey shading indicates the distribution of A-half and P-half expression respectively. The extent of each box represents the middle 50% of the ranked data with the median indicated by a horizontal bar and range by the vertical lines.

In addition to WISH, we applied a more sensitive, second revalidation procedure to a subset of these genes. PCR primers for real-time quantitative PCR (qPCR) were designed for selected genes in each category, including 9 genes from Group 4 (non-staining or no differential expression). Overall we obtained 22 pairs of primers that gave a regression coefficient of >0.99 on a standard curve of dilution and a single peak on a dissociation curve. Gene expression was calculated for 3–5 individual A- and P- half-sclerotomes (dissected as above). Notably, as with the microarray data, the highest ratios of gene expression obtained using qPCR in A- and P-halves were for *tbx18 *and *uncx4.1 *respectively. The ratios obtained for the most highly differentially-expressed transcripts within the microarray data were lower than for qPCR, suggesting that the RNA amplification in array experiments may result in underestimation of gene expression differences. Additionally, qPCR with two further control genes, *sema3a *and *spondin1*, confirmed their expression as P-restricted.*O*f the 22 genes typed by qPCR, 14 (63%) showed two-fold or greater differential expression (Figure 7, Additional File 9), while a further 3 genes show smaller but robust differences between A- and P-half-sclerotome (see Additional File 10). Most significantly, 6 of the 9 genes assigned to the 'non-staining/no differential expression' group by WISH, were re-assigned as differentially-expressed. Another 2 of these 9 genes were amongst the group of 3 genes showing small but distinct differences between A- and P-half-sclerotome. This indicates that the initial WISH screen is likely to underestimate the true proportion of differentially-expressed genes, which may actually be as high as 75%. In turn, of the original list of 175 genes, it is likely that at minimum 130 are differentially-expressed between A- and P-half-sclerotomes. Moreover, since the qPCR-positive proportion of candidates within the top 175–850 genes is 15%, the absolute number of differentially-expressed genes is likely to be substantially higher.

## Discussion

Recent studies of the molecular basis of somite development have emphasized the key roles played by the cyclical expression of genes in the presomite mesoderm, and also by counter-gradients along the A-P axis of signalling molecules [3-5]. In addition, a complex interaction of the transcription factor genes *mesp2*, *ripply2 *and *tbx6 *has been shown to establish A-P somite polarity *mesp2 *[6-11]. Our study has focussed on events that maintain polarity during sclerotome differentiation, after the initial appearance of somite polarity. Consistent with this, *mesp2*, *ripply2 *and *tbx6 *do not feature in our gene expression profiles. While numerous studies have focused on the differential expression of individual genes within the sclerotome halves, there has been no systematic attempt to characterize the overall molecular complexity in this system, and it has remained unclear whether many more differentially-expressed genes are required to maintain sclerotome polarity and differentiation. Furthermore, the molecular basis of the well-known repulsion of spinal (motor and sensory) axons by posterior-half-sclerotome has yet to be fully explained. The present study reveals a surprising degree of molecular heterogeneity between the two sclerotome halves, and highlights the potential complexity underlying an overtly simple embryonic pattern.

Our approach has been to use microarray technology with limiting quantities of starting RNA, followed by confirmation of candidacy for differential expression by whole-mount *in situ *hybridization and/or qPCR. It is interesting to compare our data with those of Buttitta *et al*. [34], who assessed gene expression in presomitic mesoderm versus newly-formed somites using much larger amounts of starting RNA. The numbers of genes identified in the two studies (several hundred) are broadly comparable, indicating that the use of much smaller starting quantities of RNA in the present study has not compromised the ability to detect gene expression differences between highly related tissues. It is of note that, when WISH is used as a secondary validation in both studies, there is a comparable reduction in the proportion of genes confirmed as differentially-expressed (50–60%). This reflects the limitations of WISH as a secondary validation procedure in these studies as the relative insensitivity of the method fails to distinguish lower levels of differential expression. While WISH gives spatial expression information in the context of embryonic anatomy, our results suggest that qPCR should be used as an additional tool to validate true differential expression. In our hands, we detect differential expression for a large number of genes originally ascribed as being not differentially-expressed by WISH.

We have uncovered a bias of differentially-expressed genes towards the P-half-sclerotome, with the over-representation of factors involved in transcriptional regulation, and of highly regulated cellular states such as phosphorylation and differentiation. Collectively, this may indicate that the development of the P-half-sclerotome is a more complex and highly regulated process compared with A-half-sclerotome. While as expected, P-half-sclerotome differentially expresses markers of bone and cartilage differentiation (e.g. *sox9*), these are notably absent from A-cells. This finding strongly supports the hypothesis that the central A-half-sclerotome does not contribute bone and cartilage to the vertebral column, promoting instead the development of the segmental spinal nerves with associated sheath elements. The neural-associated fate of central-anterior cells stands in striking contrast to that of dorsal- and ventral-anterior cells, which are known contribute to the spinous process and vertebral body respectively [2,12].

The dissected A-half sclerotomes used in our study will include a range of somite stages some of which will be expected to contain some neural crest cells that have begun to migrate within the sclerotome. Consequently, neural crest-expressed genes should be present within the A-specific genes. In agreement with this, we found well-established neural crest markers, such as *crabp1 *and *tcfap2/ap2 *[35,36], within the 57 A-half sclerotome genes. This would represent ~3.5% of the total A- specific genes. More detailed expression studies will be required to determine whether any more A-specific genes (previously not known to be expressed in neural crest cells) are also found in neural crest. Other known neural crest-specific genes, such as *sox10 *[37], have not been revealed in our analysis. This might suggest that only a small proportion of neural crest cells are found within the dissected portion of the A-half-central sclerotome within the age-range of somites in our sample, thereby diluting the expression of neural crest markers with respect to all A-specific genes. Furthermore, as our experiments examine expression differences between A and P-half-sclerotomes, neural crest markers that also have independent expression in the P-half will be necessarily be absent.

A key property of the P-half-sclerotome is to repel the outgrowth of neural crest cells and spinal nerve (motor and sensory) axons, thereby generating spinal nerve segmentation [21,25]. Candidate repellent molecules include members of the semaphorin and ephrin families, and our data have confirmed the P-specific expression of such molecules. However, several previous studies have also indicated that further P-specific repellents remain to be identified [23,27], and our results provide a starting point in this direction. Candidates warranting further investigation include the transmembrane semaphorin receptor component, PlexinA2, which is prominently expressed in the P-half-sclerotome and the cell surface heparan sulphate proteoglycan, glypican- 6 (*gpc6*). It is intriguing that N-acetylgalactosaminyltransferase 4 (*galnt4*) appears high in the candidate list (Additional File 5) since this enzyme is essential for initiating O-glycosylation of glycoproteins, and P-specific expression of an O-glycosylated peanut agglutinin receptor protein is critical for axon repulsion mediated by P-half-sclerotome cells [23].

## Conclusion

We have profiled gene expression differences that define the regional differentiation of the somite-derived sclerotome into anterior and posterior halves. Our data show that several hundred genes are differentially-expressed between the two halves. The results reveal a surprising degree of molecular heterogeneity that underlies the development of somite polarity, and indicate candidates for further investigation as regulators of somite polarity and vertebral morphogenesis, as well as repellents of spinal axon growth.

## Methods

### Sclerotome dissection and RNA isolation

Strips of ~8 consecutive somites, corresponding to the 10–18th most recently formed somites, of Theiler stage 15 (TS [38]) mouse embryos (9.5–10.25 days post-coitum, d.p.c.) were dissected into ice-cold Dulbecco's modified Eagle's medium containing Hepes. Somite strips were pinned medial-side-up with A5 insect pins, and transverse cuts used to isolate the most anterior or posterior third of the somite. A- or P-somite thirds were placed flat and all tissue surrounding the central portion of the sclerotomal mesenchyme was cut away using a 15° microfeather blade (John Weiss Ltd). A- or P-sclerotome-thirds were transferred to a 1.5 ml microcentrifuge tube containing ~30 μl RNAlater (Ambion) and total RNA was then isolated using RNeasy spin columns (Qiagen). Somite thirds rather than halves were used in order to minimize potential contamination of the samples. For consistency with published literature we refer in the text to these samples as having been isolated from somite halves.

### RNA amplification, microarray hybridization and computation

Reverse transcription and initial RNA amplification were performed using Super SMART cDNA synthesis (Clontech) as described by the manufacturer, except that the 3' SMART T7-RSAI-CDS-Oligo (dT) primer was replaced with a primer of sequence- 5'-AAGCAGTGGTATCAACGCAGAGTACGGCCAGTGAATTGTAATACGACTCACTAAGGGAGGCGGT(30)VN-3' (Sigma), and cDNA amplification was limited to 18 cycles. cDNA was purified using Nucleospin columns (BD Biosciences). Labelled, amplified RNA (aRNA) was then synthesized by *in vitro *transcription using the GeneChip^® ^IVT-Labelling kit (Affymetrix), and purified using RNAeasy spin columns (Qiagen). RNA fragmentation and hybridization to Mouse 430 GeneChip^® ^microaarrays were performed as described in the GeneChip^® ^Expression Analysis Technical Manual (Affymetrix) using an Affymetrix Hybridization Oven 640 and a GeneChip^® ^Fluidics Station 450. Arrays were scanned using a GeneChip^® ^Scanner 3000 7G, and probe intensity data (.CEL files) generated from raw scanned images (.DAT files) using the Microarray Suite software package, MAS 5.0. The 'affy' package of Bioconductor  was used to import probe intensity data into the open-source R statistical environment . Spatial artefacts were screened using the heat map function of R. Sample integrity was confirmed using MAS 5.0-generated 3'/5' probe intensity ratios for *β-actin *and *gapdh*, and RNA digestion plots were assessed using the Bioconductor 'affy' package. Model-based inter- and intra- array outlier detection was performed with dCHIP . Normalization was assessed using Bland-Altman plots within R. Background adjustment, normalization and expression measure were computed using the Bioconductor 'gcrma' package. For Welch's t-test, the t-test function of Excel was used (Microsoft). Fisher's exact scores were generated by the R Fisher.test function. Further statistical analysis was performed within R, and the Database for Annotation, Visualization and Integrated Discovery (DAVID; ).

### Antisense RNA probe synthesis

PCR primers to generate unique probe templates for antisense RNA synthesis, without significant homology to other genome sequences, were designed using a semi-automated Perl programme adapted from the Sanger Institute microarray design pipeline (Robert Andrews, Sanger Institute). Probe templates were generated by PCR from whole mouse embryo cDNA using primers with the T7 primer sequence (5'-TAATACGACTCACTATAGGGAG-3') appended to the 5' end of the reverse primer. Digoxigenin-labeled probe was synthesized from the PCR product using T7-mediated *in vitro *Megascript transcription kits (Ambion). Labelled probes were purified using G50 gel filtration columns (Amersham).

### Whole-mount mouse embryo in situ hybridization

Whole-mount *in situ *hybridization (WISH) was performed on TS15-17 (9.5–10.5 d.p.c.) mouse embryos based on a protocol from Wilkinson [39] but adapted for the InsituPro VS robotic liquid-handling system (Intavis AG). Embryos were fixed for 48 hours in 10% neutral-buffered formalin (NBF) and washed in PBS before dehydration to methanol. Embryos were bleached for 1 hour in 0.3% hydrogen peroxide/methanol before rehydration. Permeabilization with 10 μg/ml proteinase K (Roche) in PBST for 20–30 minutes was followed by quenching in 2% glycine/PBST, and fixation with 0.1% glutaraldehyde/10% NBF. After PBST washing, embryos were rinsed in hybridization buffer (0.1% triton X-100, 3× SSC, 1% Roche blocking powder, 50% formamide, 0.1% CHAPS, 5 mM EDTA, 50 μ/ml heparin) prior to pre-hybridization at 62°C for 6 hours in fresh buffer. Samples were then hybridised for 18 hours with the addition of digoxigenin-labeled antisense probe (1 μg/ml). Post-hybridization embryos were washed in 0.1% CHAPS, 2× SSC, 50% formamide followed by 0.1% CHAPS, 0.2× SSC. After room temperature washes in PBST, embryos were blocked in 20% goat serum/PBST followed by antibody incubation (1/1000 Roche alkaline phosphatase-conjugated sheep anti-digoxigenin fab fragments/10% goat serum/PBST) for 6 hours. Embryos were washed 10 times in PBST, and 5 times in 100 mM Tris-HCl (pH 9.0), 100 mM NaCl, 0.1% triton X-100 before colour development using BCIP/NBT (Roche).

### Quantitative real-time PCR

Quantitative real-time PCR (qPCR) was performed using an efficiency-corrected ΔCt method using SYBR^® ^green on an ABI 7500 Prism platform. Primers were designed to span intron-exon boundaries using the open-source PerlPrimer package (, see Additional File 11). Only primer pairs with a correlation coefficient of > 0.99 in a standard curve of Ct values plotted against dilution, and a single peak on a dissociation curve, were used. Gene expression measures were calculated from the geometric mean of 4–5 replicate readings for each sample. Outliers with more than one standard deviation from the mean Ct and replicates showing more than 5% standard deviation from the mean Ct were excluded. Gene expression for the gene of interest (GOI) was normalized relative to β-actin using E_GOI_^(CtGOI)^/E_β-actin_^(Ct β-actin)^, where E = efficiency of amplification and is derived from the standard curve (10^(-1/slope)^). To ensure the identity of anterior- or posterior-half-sclerotomes, *uncx4.1 *(a posterior-specific marker) expression relative to β-actin was determined. Samples showing ΔΔCt > 10 or <8 were considered as correctly dissected anterior or posterior samples respectively. The relative expression of a GOI was calculated from the RQ (relative quality) ratios derived from the mean anterior and posterior ΔΔCt values. The lowest values of ΔΔCt (highest concentration) were used to calculate the RQ ratios for the highest level of differential expression between anterior and posterior samples. Normalized graphical representation of qPCR data (see Figure 7 & Additional File 10) were generated by calculating E_β-actin_^(Ct β-actin)^/E_GOI_^(Ct GOI) ^for each sample of a GOI, and calculating all values as a percentage of the highest value (anterior or posterior) obtained for that gene.

## Abbreviations

A: anterior; ABI: Applied Biosystems; A-P: anteroposterior; aRNA: amplified ribonucleic acid; BCIP: 5-Bromo-4-chloro-3-indolyl phosphate; BLAST: Basic Local Alignment Search Tool; cDNA: complementary deoxyribonucleic acid; CHAPS: 3-[(3-Chlolamidopropyl) dimethyl-ammonio-1-propane sulfonate; DAVID: Database for Annotation, Visualization and Integrated Discovery; dpc: days post-cotium; DNA: deoxyribonucleic acid; D: dorsal; e: embryonic days post-cotium; EDTA: ethylenediaminetetraacetic acid; esm: epithelial somite; Fab: fragment antigen binding; FGF: fibroblast growth factor; GO: Gene Ontology; GOI: gene of interest; ivd: intervertrebral disc; ivf: intervertebral foraminae; KEGG: Kyoto Encyclopedia of Genes and Genomes; PSM: presomitic mesoderm; MAS: Microarray Suite software package; NBF: neutral-buffered formalin; NBT: nitro blue tetrazolium chloride; P: posterior; PBS: phosphate-buffered saline; PBST: phosphate-buffered saline with triton; PCR: polymerase chain reaction; PFAM: Protein families database; PNS: peripheral nervous system; qPCR: quantitative polymerase chain reaction; RNA: ribonucleic acid; SAM: Significance Analysis of Microarrays; sp: spinous process; SSC: standard citrate saline; TS: Theiler stage; V: ventral; WISH: whole mount *in situ *hybridization; vb: vertebral body.

## Competing interests

The authors declare that they have no competing interests.

## Authors' contributions

DT and RJK conceived the study. DT supervised and guided the project. DSTH performed, designed the experiments and carried out data analysis in fulfilment of a PhD for the University of Cambridge. RJK performed the dissections. DT, RJK and DSTH prepared the manuscript.

## Supplementary Material

Additional file 1 **Affymetrix identifiers and ranks for 11 known differentially-expressed sclerotome genes**. Affymetrix identifiers of genes known to be differentially-expressed in A- or P-half-sclerotome. Multiple entries for the same gene reflect the transcripts represented on the Affymetrix GeneChip (26 transcripts corresponding to 11 genes, bold indicating the highest probability transcript for each gene). The probability of differential gene expression by Fisher's analysis, and the overall rank in array data are also listed. Click here for file

Additional file 2 **Further clustering of individual array experiments**. Dendrograms for agglomerative hierarchical clustering of individual array hybridization experiments showing that arrays do not separate into A- or P-clusters when analysed against: (i) the whole genome (45,101 GeneChip transcripts); (ii) sclerotome-expressed genes without differential A-P expression (*pax1, pax9*, *scleraxis*); (iii) general somite markers (*cdx1*, *etv5*, *fgf6, foxc1*, *foxc2, pax1*, *pax3*, *pax7*, *pax9*, *myoD*, *myogenin*, *myf5*, *scleraxis*, *sfrp2); *or (iv) house-keeping genes (*β-actin *and *gapdh*). Click here for file

Additional file 3 **Distribution of known transcripts amongst all rank-ordered transcripts**. (i) The distribution of 11 transcripts, with known differential expression between A- and P- half-sclerotome, shows that while most are evenly distributed across the rank-ordered transcripts, more than one-third fall within the cell representing the highest statistical certainty of differential expression (see Additional File 1 for transcript identifiers). (ii) In contrast, the distribution of genes with no differential expression within the sclerotome shows no such peak. Histograms were generated using the *hist *function of R with a cell number of 50. Click here for file

Additional file 4 **Statistical significance of differential gene expression in the array data**. The graph shows Fisher's exact score from Figure 4 for the range of 0–1500 rank-ordered transcripts. See legend to Figure 4 for further description. The blue line corresponds to p = 10^-7^. Differential expression is highly significant for the top ~650 transcripts. Click here for file

Additional file 5 **Differentially-expressed genes between A- and P-half-sclerotome (genes 1–175)**. Table showing rank order of probability of differentially expression in A- or P-half-sclerotome for the top 175 transcripts as determined by t-test. Click here for file

Additional file 6 **Differentially-expressed genes between A- and P-half-sclerotome (genes 176–850)**. Table showing rank order of probability of differentially expression in A- or P-half-sclerotome for transcripts 176–850 as determined by t-test. Click here for file

Additional file 7 **Functional categorization of differentially-expressed genes**. Table showing terms for biological processes that show a significant probability of enrichment relative to the genome for the first 175 genes as determined by DAVID. Click here for file

Additional file 8 **Whole-mount *in situ *hybridization of additional sclerotome differentially-expressed candidates**. A further 44 genes in addition to those described in Figure 6 were typed by *in situ *hybridization for differential expression between A- and P-half-sclerotomes. Expression patterns were classified into 4 groups as for Figure 6 (see also Table 1). Group 1 (unambiguous differential expression, green text): *flrt2, meox1, nmyc1, uncx4.1, igfbp5, lef1; *Group 2 (very likely to be differentially-expressed, yellow text): *efnb2, acpl2, mtdh, rbp1, rps6, sema3a, fap; *Group 3 (likely to be differentially-expressed, blue text): *anapc4, ets2, fgfr1op, gpr150, trappc6b, rab28, tpr, nm_172870, q8c5e4*. Group 4 (non-staining or no differential expression, brown text): *apg5l, btc, cnot, d17wsu104e, lxn, narg1, nm_028130, enh, nm_027740, q8cak8, st13, tbc1d24, tcfa2b, zc3h6, cd44, cdc42, col4a5, dcc, ddx3y, eif4e, galg1, hexb*. Click here for file

Additional file 9 **Expression measures for qPCR analysis**. Table showing the qPCR expression measures of a selection of candidate differentially-expressed genes between A- and P-half sclerotome. Genes in red are false-positive (FP) with no indication of differential expression. * Expression is highly dynamic within the dissected P-half-sclerotomes. While the average differences between A and P are <2, there is a greater than 2-fold difference between the highest levels of expression in P relative to A. # Genes showing small but distinct differences between A- and P-half-sclerotome. This may account for their initial categorization as non-differentially-expressed by whole-mount *in situ *hybridization. Click here for file

Additional file 10 **Further qPCR expression analysis of differential expression**. qPCR was used to show differential expression of 3 genes (*mospd2, nedd4, apg5l*) showing small but robust P-half-sclerotome enrichment (See Additional File 9). In contrast, 4 candidate differentially-expressed genes (*fgf1rop, mtdh, st13, wnt5A*) appear to be false-positives as qPCR provides no evidence for differential expression. Data are displayed as in Figure 7 legend. Click here for file

Additional file 11 **Primers used for qPCR analysis**. Table indicating the primer sequences used for qPCR analysis of candidate differentially-expressed genes. Click here for file
