# Supplementary material for: Extensive molecular differences between anterior- and posterior-half-sclerotomes underlie somite polarity and spinal nerve segmentation
Source: BMC Dev Biol. 2009 May 22;9:30. doi: 10.1186/1471-213X-9-30 (PMC2693541; doi:10.1186/1471-213X-9-30)
Supplement: Additional file 1 — Affymetrix identifiers and ranks for 11 known differentially-expressed sclerotome genes. Affymetrix identifiers of genes known to be differentially-expressed in A- or P-half-sclerotome. Multiple entries for the same gene reflect the transcripts represented on the Affymetrix GeneChip (26 transcripts corresponding to 11 genes, bold indicating the highest probability transcript for each gene). The probability of differential gene expression by Fisher's analysis, and the overall rank in array data are also listed. [file 1471-213X-9-30-S1.doc]

Additional File 1

Affymetrix identifiers and ranks for 11 known differentially-expressed sclerotome genes

|  | **Affymetrix ID** | **Name** | **Orientation** | **Description** | **Rank** | **p (Fisher’s)** |
| --- | --- | --- | --- | --- | --- | --- |
| 1 | 1419633_at | **uncx4.1** | Posterior | Unc4.1 homeobox (C. elegans) | 17 | 0.009748 |
| 2 | 1417595_at | **meox1** | Posterior | mesenchyme homeobox 1 | 18 | 5.24E-05 |
| 3 | 1417155_at | **nmyc1** | Posterior | neuroblastoma myc-related oncogene 1 | 35 | 1.38E-06 |
| 4 | 1429974_at | **tbx18** | Anterior | T-box18 | 109 | 7.23E-07 |
| 5 | 1420416_at | **sema3a** | Posterior | sema domain, immunoglobulin domain (Ig), short basic domain, secreted, (semaphorin) 3A | 144 | 4.06E-08 |
| 6 | 1419639_at | **efnb2** | Posterior | ephrin B2 | 172 | 1.87E-09 |
| 7 | 1449871_at | **tbx18** | Anterior | T-box18 | 664 | 3.21E-07 |
| 8 | 1419638_at | efnb2 |  | ephrin B2 | 684 | 2.37E-08 |
| 9 | 1449865_at | sema3a |  | sema domain, immunoglobulin domain (Ig), short basic domain, secreted, (semaphorin) 3A | 4168 | 0.001631 |
| 10 | 1420508_at | **sema3f** | Posterior | sema domain, immunoglobulin domain (Ig), short basic domain, secreted, (semaphorin) 3 F | 4243 | 0.000536 |
| 11 | 1440069_at | efnb2 |  | ephrin B2 | 8648 | 0.03663 |
| 12 | 1424050_s_at | **fgfr1** | Anetrior | fibroblast growth factor receptor 1 | 15875 | 0.3544 |
| 13 | 1422923_at | **fgf3** | Posterior | fibroblast growth factor 3 | 21322 | 0.8621 |
| 14 | 1436551_at | fgfr1 |  | fibroblast growth factor receptor 1 | 22930 | 0.7383 |
| 15 | 1420417_at | sema3a |  | sema domain, immunoglobulin domain (Ig), short basic domain, secreted, (semaphorin) 3A | 24037 | 0.7409 |
| 16 | 1425922_a_at | nmyc1 |  | neuroblastoma myc-related oncogene 1 | 27694 | 0.8738 |
| 17 | 1425923_at | nmyc1 |  | neuroblastoma myc-related oncogene 1 | 28503 | 0.8744 |
| 18 | 1451550_at | **ephb3** | Posterior | Eph receptor B3 | 30670 | 0.8773 |
| 19 | 1424415_s_at | spon1 |  | spondin 1, (f-spondin) extracellular matrix protein | 31604 | 0.8785 |
| 20 | 1451342_at | spon1 |  | spondin 1, (f-spondin) extracellular matrix protein | 32351 | 0.7632 |
| 21 | 1449548_at | efnb2 |  | ephrin B2 | 37317 | 1 |
| 22 | 1425911_a_at | fgfr1 |  | fibroblast growth factor receptor 1 | 38951 | 1 |
| 23 | 1442613_at | spon1 |  | spondin 1, (f-spondin) extracellular matrix protein | 39876 | 0.8863 |
| 24 | 1449549_at | efnb2 |  | ephrin B2 | 41546 | 0.8875 |
| 25 | 1425840_a_at | sema3f |  | sema domain, immunoglobulin domain (Ig), short basic domain, secreted, (semaphorin) 3 F | 45057 | 1 |
