# Supplementary figures and images for: Extensive molecular differences between anterior- and posterior-half-sclerotomes underlie somite polarity and spinal nerve segmentation
Source: BMC Dev Biol. 2009 May 22;9:30. doi: 10.1186/1471-213X-9-30 (PMC2693541; doi:10.1186/1471-213X-9-30)

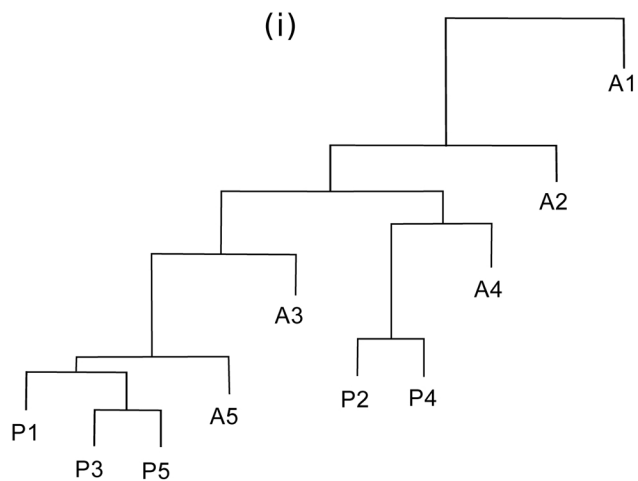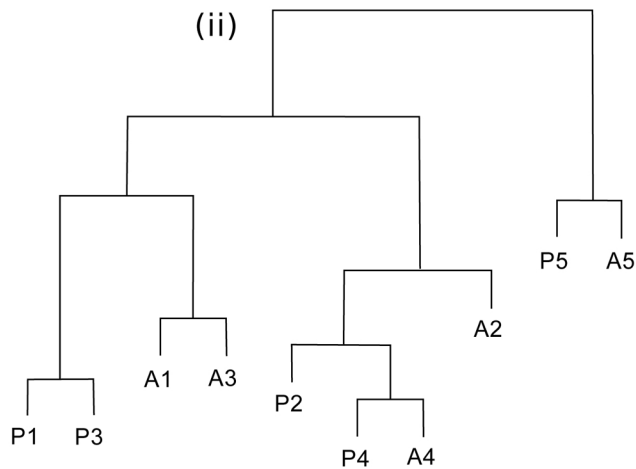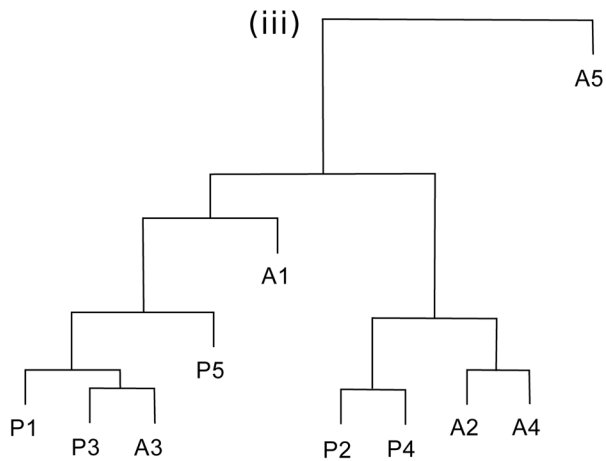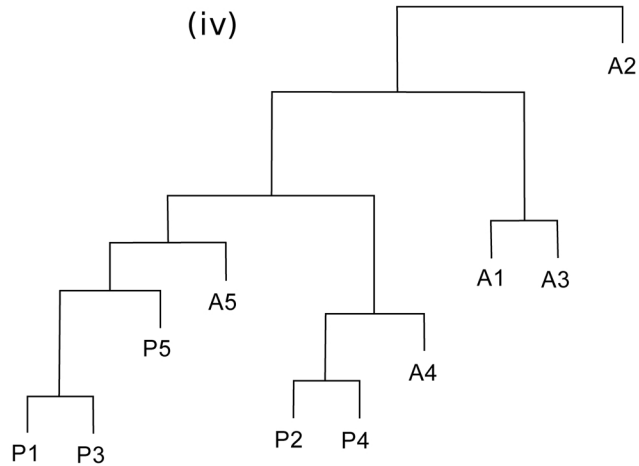

Supplement: Additional file 2 — Further clustering of individual array experiments. Dendrograms for agglomerative hierarchical clustering of individual array hybridization experiments showing that arrays do not separate into A- or P-clusters when analysed against: (i) the whole genome (45,101 GeneChip transcripts); (ii) sclerotome-expressed genes without differential A-P expression (pax1, pax9, scleraxis); (iii) general somite markers (cdx1, etv5, fgf6, foxc1, foxc2, pax1, pax3, pax7, pax9, myoD, myogenin, myf5, scleraxis, sfrp2); or (iv) house-keeping genes (β-actin and gapdh). [file 1471-213X-9-30-S2.pdf]

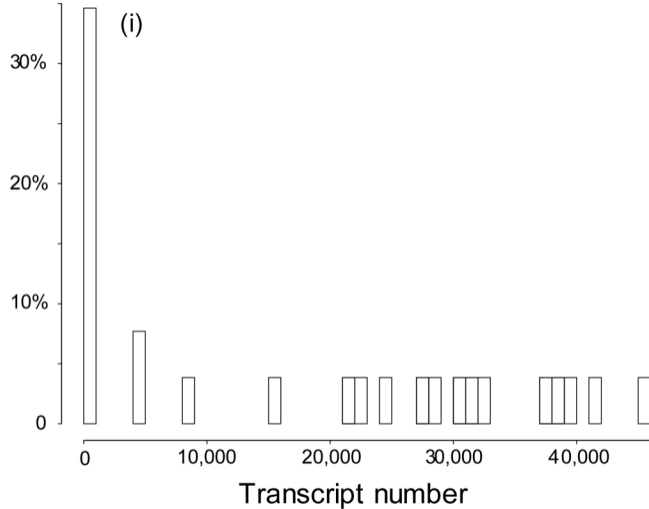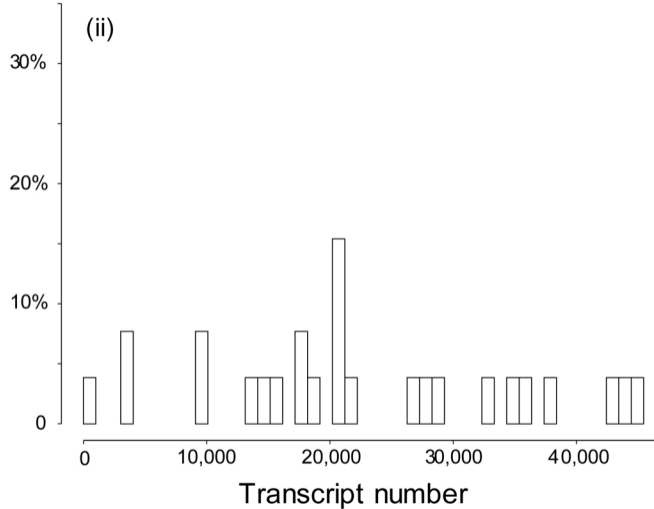

Supplement: Additional file 3 — Distribution of known transcripts amongst all rank-ordered transcripts. (i) The distribution of 11 transcripts, with known differential expression between A- and P- half-sclerotome, shows that while most are evenly distributed across the rank-ordered transcripts, more than one-third fall within the cell representing the highest statistical certainty of differential expression (see Additional File 1 for transcript identifiers). (ii) In contrast, the distribution of genes with no differential expression within the sclerotome shows no such peak. Histograms were generated using the hist function of R with a cell number of 50. [file 1471-213X-9-30-S3.pdf]

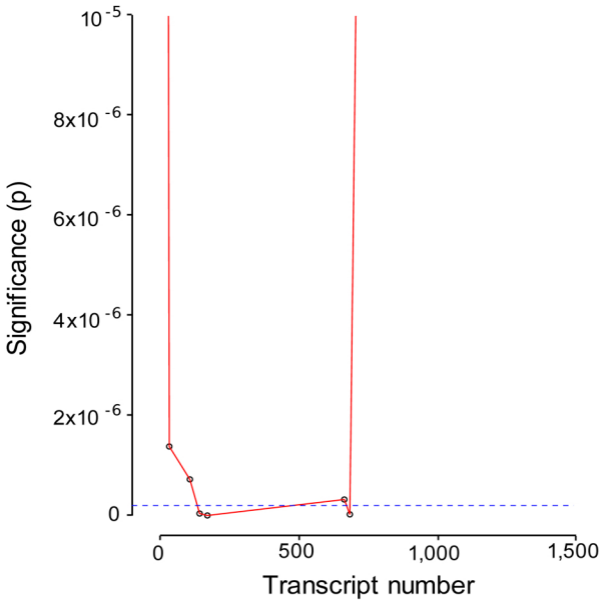

Supplement: Additional file 4 — Statistical significance of differential gene expression in the array data. The graph shows Fisher's exact score from Figure 4 for the range of 0–1500 rank-ordered transcripts. See legend to Figure 4 for further description. The blue line corresponds to p = 10-7. Differential expression is highly significant for the top ~650 transcripts. [file 1471-213X-9-30-S4.pdf]

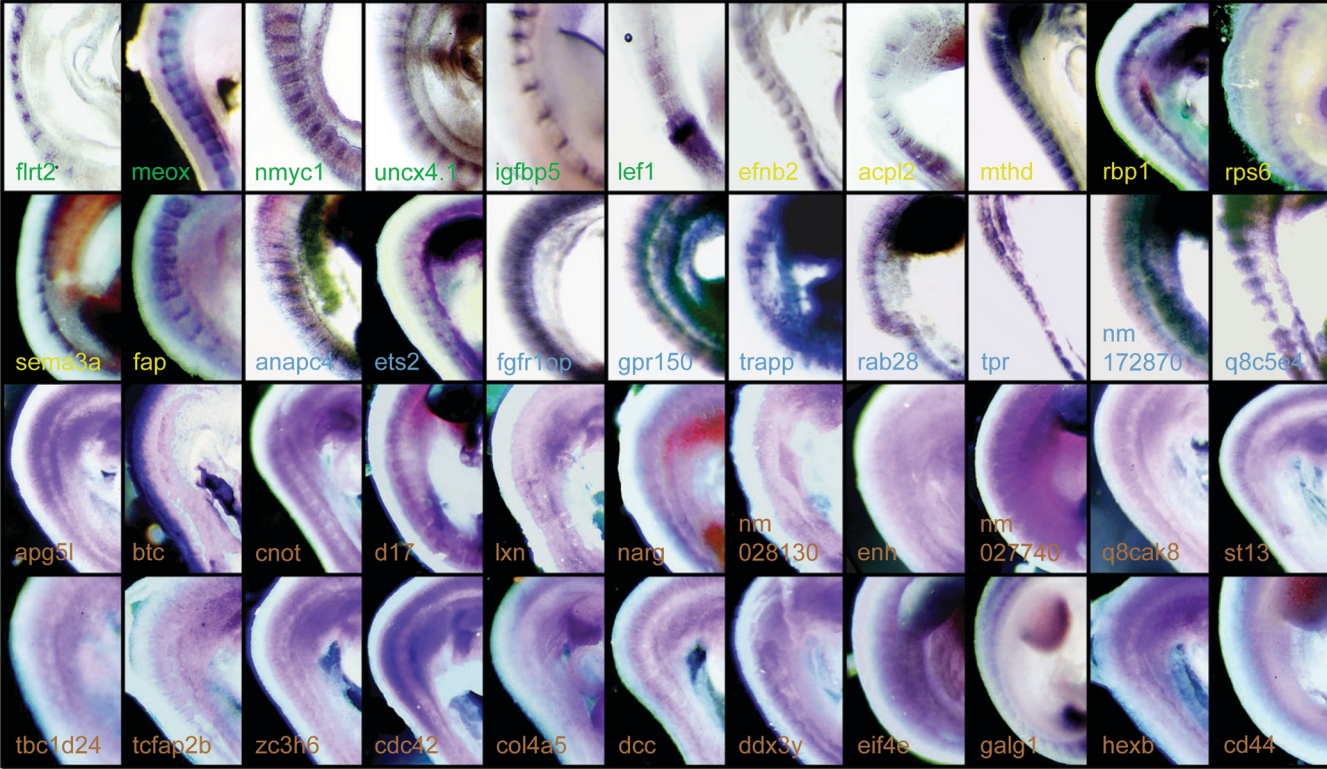

Supplement: Additional file 8 — Whole-mount in situ hybridization of additional sclerotome differentially-expressed candidates. A further 44 genes in addition to those described in Figure 6 were typed by in situ hybridization for differential expression between A- and P-half-sclerotomes. Expression patterns were classified into 4 groups as for Figure 6 (see also Table 1). Group 1 (unambiguous differential expression, green text): flrt2, meox1, nmyc1, uncx4.1, igfbp5, lef1; Group 2 (very likely to be differentially-expressed, yellow text): efnb2, acpl2, mtdh, rbp1, rps6, sema3a, fap; Group 3 (likely to be differentially-expressed, blue text): anapc4, ets2, fgfr1op, gpr150, trappc6b, rab28, tpr, nm_172870, q8c5e4. Group 4 (non-staining or no differential expression, brown text): apg5l, btc, cnot, d17wsu104e, lxn, narg1, nm_028130, enh, nm_027740, q8cak8, st13, tbc1d24, tcfa2b, zc3h6, cd44, cdc42, col4a5, dcc, ddx3y, eif4e, galg1, hexb. [file 1471-213X-9-30-S8.pdf]

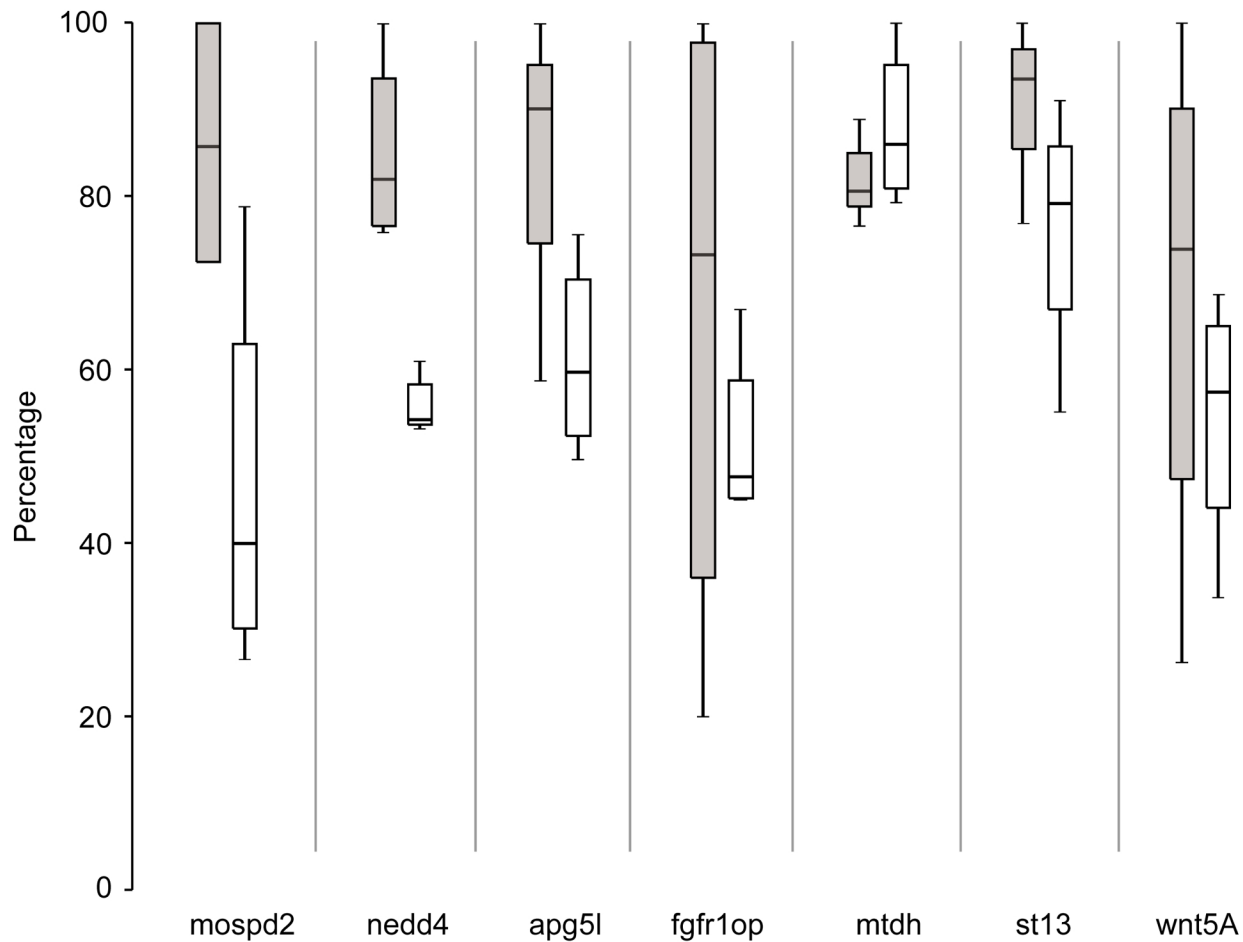

Supplement: Additional file 10 — Further qPCR expression analysis of differential expression. qPCR was used to show differential expression of 3 genes (mospd2, nedd4, apg5l) showing small but robust P-half-sclerotome enrichment (See Additional File 9). In contrast, 4 candidate differentially-expressed genes (fgf1rop, mtdh, st13, wnt5A) appear to be false-positives as qPCR provides no evidence for differential expression. Data are displayed as in Figure 7 legend. [file 1471-213X-9-30-S10.pdf]
