# Supplementary material for: Extensive molecular differences between anterior- and posterior-half-sclerotomes underlie somite polarity and spinal nerve segmentation
Source: BMC Dev Biol. 2009 May 22;9:30. doi: 10.1186/1471-213X-9-30 (PMC2693541; doi:10.1186/1471-213X-9-30)
Supplement: Additional file 9 — Expression measures for qPCR analysis. Table showing the qPCR expression measures of a selection of candidate differentially-expressed genes between A- and P-half sclerotome. Genes in red are false-positive (FP) with no indication of differential expression. * Expression is highly dynamic within the dissected P-half-sclerotomes. While the average differences between A and P are <2, there is a greater than 2-fold difference between the highest levels of expression in P relative to A. # Genes showing small but distinct differences between A- and P-half-sclerotome. This may account for their initial categorization as non-differentially-expressed by whole-mount in situ hybridization. [file 1471-213X-9-30-S9.doc]

Additional File 9

**Expression measures for qPCR analysis**

| **Gene** | **ISH Catagory** | **Orientation** | **Mean P** | **Mean A** | **Ratio of mean values** | **Ratio of maximum values** |
| --- | --- | --- | --- | --- | --- | --- |
| arhg * | Group 4 | Posterior | 73.70 | 41.08 | 1.79 | 2.10 |
| dach1 | no WISH probe | Posterior | 87.55 | 29.07 | 3.01 | 2.25 |
| dcc # | Group 4 | Anterior | Only detected in A-sclerotome | | | |
| enh * | Group 4 | Posterior | 71.79 | 37.57 | 1.91 | 2.39 |
| gpc6 | Group 1 | Posterior | 77.13 | 14.85 | 5.19 | 5.57 |
| plxnA2 | Group 1 | Posterior | 82.61 | 16.08 | 5.14 | 5.03 |
| q8kac8 | Group 4 | Anterior | 7.31 | 75.44 | 10.31 | 9.25 |
| rps6 | Group 2 | Posterior | 55.00 | 23.23 | 2.37 | 2.41 |
| sema3A | Group 2 | Posterior | 53.74 | 6.51 | 8.25 | 11.21 |
| slit2 | Group 4 | Posterior | 67.27 | 25.11 | 2.68 | 2.99 |
| spon1 | P-sclerotome control gene | Posterior | 89.17 | 41.77 | 2.13 | 2.17 |
| tbx18 | no WISH probe | Anterior | 2.50 | 77.54 | 30.98 | 6.24 |
| tcfap2b | Group 4 | Anterior | 16.03 | 52.61 | 3.28 | 13.45 |
| tgfbr2 | no WISH probe | Posterior | 94.84 | 47.66 | 1.99 | 1.13 |
| trappc6b | no WISH probe | Posterior | 81.19 | 25.97 | 3.13 | 2.96 |
| xlr4 | no WISH probe | Posterior | 100.00 | 8.06 | 12.40 | 6.98 |
| apg5l * | Group 4 | Posterior | 83.16 | 61.45 | 1.35 | 1.32 |
| mospd2 # | Group 3 | Posterior | 86.24 | 46.63 | 1.85 | 1.27 |
| nedd4 # | Group 4 | Posterior | 85.21 | 55.97 | 1.52 | 1.64 |
| fgfr1op | Group 3 | FP | 66.92 | 52.14 | 1.28 | 1.49 |
| mtdh | Group 2 | FP | 82.25 | 88.08 | 1.07 | 1.12 |
| st13 | Group 4 | FP | 90.33 | 76.41 | 1.18 | 1.10 |
| wnt5a | no WISH probe | FP | 68.79 | 54.61 | 1.26 | 1.45 |
