# Supplementary material for: Extensive molecular differences between anterior- and posterior-half-sclerotomes underlie somite polarity and spinal nerve segmentation
Source: BMC Dev Biol. 2009 May 22;9:30. doi: 10.1186/1471-213X-9-30 (PMC2693541; doi:10.1186/1471-213X-9-30)
Supplement: Additional file 11 — Primers used for qPCR analysis. Table indicating the primer sequences used for qPCR analysis of candidate differentially-expressed genes. [file 1471-213X-9-30-S11.doc]

Additional File 11

**Primers used for qPCR Analysis**

| actB | GAAGATTAAGATCATTGCTCCC | GCCTGACTCATCATACTCCT |
| --- | --- | --- |
| gpc6 | ACAGCAGATCATGGCTCTC | CTGGATTCATCGCTTGTGTC |
| pax1 | AGTGAATGGACTCGAGAAACCT | GGAAGCCGACTGAGTATATTTAATGTC |
| pax9 | GAATGGATTGGAGAAGGGAG | AGCACTGTAGGTCATGTAGG |
| plxnA2 | GCTGAAGAAGATTCGAGCTG | CAGAGACCATCTCATACTGGA |
| sema3A | TAGACGGTGAGTTGTACTCTG | ATCTAGGATCATTGAGCCACC |
| slit2 | CAACCGTCTGAGATGTATCC | CTCCAATCGCTAAGTGTGAC |
| spon1 | TCAAGCAAGTTGCTGAACTG | GACTTCATCACTCTGTTGTCG |
| tbx18 | GAATCAGCAGATTACTCGCC | CTCCAGAATGCGTATGACTC |
| uncx4.1 | GTTCAATGAGAGCCACTACC | TTGGAACCAGACCTGAACTC |
| wnt5a | ACAACATCGACTATGGCTACC | TGTATACTGTCCTACGGCCT |
| apg5l | ATGTCGTGTATGAAAGAAGCTG | ACTGGTCAAATCTGTCATTCTG |
| arhgap5 | ACACATCTAAACAGGGTTAGTC | TTAGTGGTAGACAGAAACTCTC |
| dach1 | CAGATGAACCACCTTAGCAC | GAACACGCTCCTTAATAACAG |
| dcc | TAAATCACCCTTCCAACCTC | TTGCTTCCTCCCACTATCTG |
| enh | GTGAGACAGACTACTACGCC | TATCATGCCAAGTGTATCCCA |
| fgfr1op | TCCTCCATCAAAGTCACCTG | CTGCTAGTCTTCTTACTTGGG |
| mospd2 | GACTCAATAGCATAGACATGGA | TCCAAGGCATATCAAAGATCAC |
| mtdh | GCCAGTTTCTCAGTCTACCA | GAAGACAAACCATTTAACCCAG |
| nedd4 | TCTGCTACGGATAATTACACCC | CTTGCCATGATAAACTGCCA |
| q8kac8 | ACATCAGCAACAAGATAGGAC | ATAGTCCAGTGGTCTTTCAG |
| st13 | AAATGCAGAGATAACCGAGGA | GTACAGAATAGCCAAGCGAG |
| tcfap2b | CGGGATTTCGGGTATATTTGTG | TCTTTACAAAGTTGCTTGGTGG |
| tgfbr2 | CAAGTCGGATGTGGAAATGG | AAATGTTTCAGTGGATGGATGG |
| trappc6b | GGACTGATAGAAAGGTTTACGA | TATATGCCCTGATGATTTGTCC |
| xlr4 | CGACACTTGAAGAATCTACAG | ATTGAACACCATCCTTTGCT |
